# Development and Validation of Multivariable Prediction Algorithms to Estimate Future Walking Behavior in Adults: Retrospective Cohort Study

**DOI:** 10.2196/44296

**Published:** 2023-01-27

**Authors:** Junghwan Park, Gregory J Norman, Predrag Klasnja, Daniel E Rivera, Eric Hekler

**Affiliations:** 1 Herbert Wertheim School of Public Health and Human Longevity Science University of California, San Diego La Jolla, CA United States; 2 Center for Wireless & Population Health Systems, Calit2’s Qualcomm Institute University of California, San Diego La Jolla, CA United States; 3 The Design Lab University of California, San Diego La Jolla, CA United States; 4 Ministry of Health and Welfare Korean National Government Sejong Republic of Korea; 5 Department of Global Access and Evidence Dexcom Inc. San Diego, CA United States; 6 School of Information University of Michigan Ann Arbor, MI United States; 7 Control Systems Engineering Laboratory School for Engineering of Matter, Transport, and Energy Arizona State University Tempe, AZ United States

**Keywords:** mobile health, mHealth, physical activity, walk, prediction, classification, multilayered perceptron, microrandomized trial, MRT, just-in-time adaptive intervention, JITAI, prevention, female, development, validation, application

## Abstract

**Background:**

Physical inactivity is associated with numerous health risks, including cancer, cardiovascular disease, type 2 diabetes, increased health care expenditure, and preventable, premature deaths. The majority of Americans fall short of clinical guideline goals (ie, 8000-10,000 steps per day). Behavior prediction algorithms could enable efficacious interventions to promote physical activity by facilitating delivery of nudges at appropriate times.

**Objective:**

The aim of this paper is to develop and validate algorithms that predict walking (ie, >5 min) within the next 3 hours, predicted from the participants’ previous 5 weeks’ steps-per-minute data.

**Methods:**

We conducted a retrospective, closed cohort, secondary analysis of a 6-week microrandomized trial of the *HeartSteps* mobile health physical-activity intervention conducted in 2015. The prediction performance of 6 algorithms was evaluated, as follows: logistic regression, radial-basis function support vector machine, eXtreme Gradient Boosting (XGBoost), multilayered perceptron (MLP), decision tree, and random forest. For the MLP, 90 random layer architectures were tested for optimization. Prior 5-week hourly walking data, including missingness, were used for predictors. Whether the participant walked during the next 3 hours was used as the outcome. K-fold cross-validation (K=10) was used for the internal validation. The primary outcome measures are classification accuracy, the Mathew correlation coefficient, sensitivity, and specificity.

**Results:**

The total sample size included 6 weeks of data among 44 participants. Of the 44 participants, 31 (71%) were female, 26 (59%) were White, 36 (82%) had a college degree or more, and 15 (34%) were married. The mean age was 35.9 (SD 14.7) years. Participants (n=3, 7%) who did not have enough data (number of days <10) were excluded, resulting in 41 (93%) participants. MLP with optimized layer architecture showed the best performance in accuracy (82.0%, SD 1.1), whereas XGBoost (76.3%, SD 1.5), random forest (69.5%, SD 1.0), support vector machine (69.3%, SD 1.0), and decision tree (63.6%, SD 1.5) algorithms showed lower performance than logistic regression (77.2%, SD 1.2). MLP also showed superior overall performance to all other tried algorithms in Mathew correlation coefficient (0.643, SD 0.021), sensitivity (86.1%, SD 3.0), and specificity (77.8%, SD 3.3).

**Conclusions:**

Walking behavior prediction models were developed and validated. MLP showed the highest overall performance of all attempted algorithms. A random search for optimal layer structure is a promising approach for prediction engine development. Future studies can test the real-world application of this algorithm in a “smart” intervention for promoting physical activity.

## Introduction

Physical inactivity is associated with numerous chronic diseases, including cancer, cardiovascular disease, type 2 diabetes [1-3], increased health care expenditure [4], and preventable, premature deaths [4]. Insufficient physical activity (PA) cost $53.8 billion worldwide in 2013. Clinical guidelines indicate 8000-10,000 steps per day [5]; nevertheless, the majority of Americans fall short of this goal [6].

In order to increase the level of PA, more than 300 commercial mobile apps have been developed [7]. The recent development of information technologies enabled mobile apps to deliver behavior change support when the users need this the most or when the utility (eg, how much the amount of PA was increased by the in-app notification) is predicted to be high. This new, promising type of intervention is called a just-in-time adaptive intervention (JITAI) [8].

JITAIs are not widely used (eg, 2.2% in 2018 [7]) by commercially available apps. However, it has been shown that JITAIs have the capacity to improve adherence and efficacy [9-11]. In addition, health behavior theories that commonly work as theoretical foundations for JITAIs [9], including social cognitive theory [12] and goal setting theory [13], emphasize the importance of timely feedback and anticipatory intervention [12,14-16]. Adaptation to individual, time-varying needs is theorized to be an effective strategy [14] for implementing time-accurate feedback and anticipatory intervention [16]. Since the opportunity window to intervene depends on the individual’s environment, a fully automatic, predictive algorithm that can be run repeatedly is one of the key components of JITAI apps [14]. Thus, developing accurate algorithms to empower JITAIs to promote PA is a central task in overall JITAI development.

Prior JITAI studies used pure randomizations [17], condition-triggered Boolean logic [18,19], a combination of manually designed logics [20], or models that reveal the mathematical relationships between input factors and the behavior (eg, system identification [21]) so that researchers could understand which factors are predictive of the behavior. In this study, the models were evaluated mainly focusing on predictive accuracy rather than explainability [22]. Time series data of walking behavior (ie, steps per minute) measured by a wearable sensor was used to predict future walking behavior. Multiple algorithms were compared using various metrics, including accuracy, Mathew correlation coefficient (MCC), sensitivity, and specificity. If these algorithms can be produced, it would be a critical step toward JITAIs that are cost-efficient and fully autonomous (ie, without human couch interventions), and thus, it could be a valuable part of overall approaches for improving population health. To ensure the model's cost-efficiency and real-time usage feasibility, the training computation time was measured in the standardized computing environment.

## Methods

### Source of Data

This study used the deidentified Jawbone walking data (ie, steps per minute) from the *HeartSteps* study [23], conducted in the United States from August 2015 to January 2016.

### Ethical Considerations

The original study [23] was approved by the University of Michigan Social and Behavioral Sciences Institutional Review Board (HUM00092845) for data collection. As the data in this study were deidentified prior to being provided, the study was deemed as nonhuman subject research by the University of California, San Diego Institutional Review Board. This study adhered to the TRIPOD (Transparent Reporting of a multivariable prediction model for Individual Prognosis or Diagnosis) statement on reporting development and validation of the multivariate predictive models [24] (Multimedia Appendix 1).

### Study Design and Data Processing Protocol

#### Exclusion and Data Transformation

Minute-by-minute walking data (ie, number of steps per minute) were preprocessed in the following three steps: (1) excluded the participants who have the data of less than 10 days, (2) excluded the data if the participant was inactive (ie, 0 step per minute) or partially active (ie, less than 60 steps per minute) during the minute, and (3) excluded short walks lasted less than 5 minutes. Then, walk data were used to decide whether the participant was active or not during the hour. If there was one or more walks (ie, more than 5 consecutive walking minutes) during the hour, it was marked as an “active hour.” Then, the data were transformed to fit the machine learning algorithms (ie, from the time-series DataFrame objects of *Pandas* library to numerical array objects containing vector objects of *NumPy* library).

#### Training of Machine Learning Algorithms

**Figure 1:**
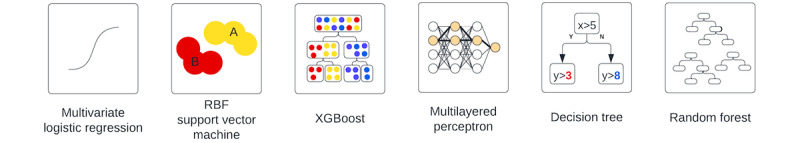
Brief algorithm descriptions of classification models. RBF: radial basis function.

The hourly walk data of the 5 prior weeks were used to predict the outcome (ie, whether the participant will walk or not during the next 3 hours). The following 6 sets of algorithms were used: logistic regression, radial basis function support vector machine [25], XGBoost [26], multilayered perceptron [27], decision tree, and random forest [28] (Figure 1). We used the implementation of the open-source projects named “scikit-learn” [29], Keras [30], XGBoost [26,31], and “Sci-Keras” [32] for each algorithm.

#### Target Imbalance

Due to sleeping hours and sedentary hours, nonactive hours usually outnumbered active hours. In machine learning algorithms, the phenomena are called “target imbalance” [33,34]. They usually critically reduce the performance of the prediction algorithm. Thus, in this study, we randomly sampled the nonactive hours to attain the same number as that of active hours.

#### K-fold Validation

**Figure 2:**
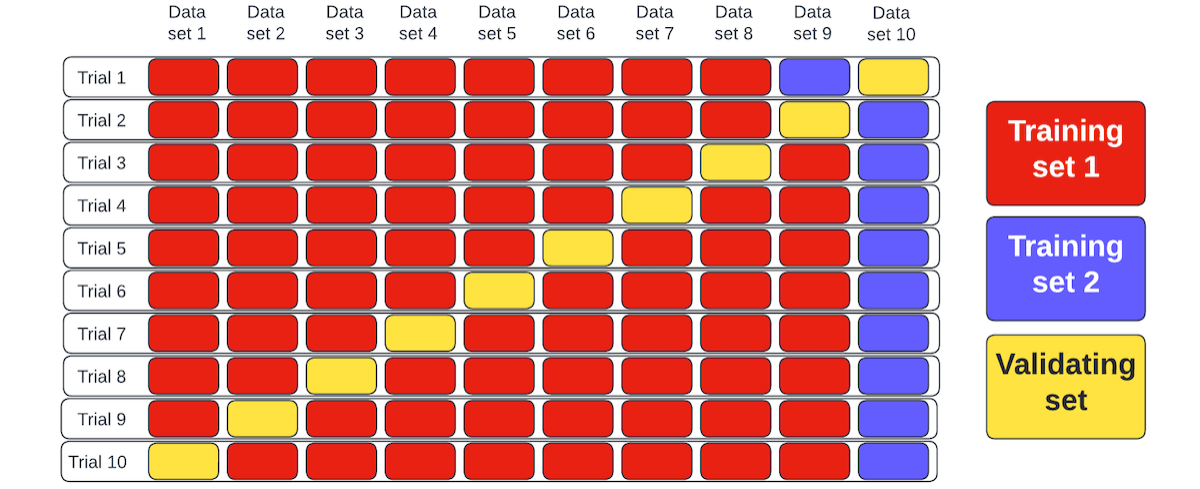
Brief description of K-fold validation method (eg, K=10).

After balancing the targets, the data were shuffled to perform K-fold validation [35] (Figure 2). We used K=10 in this study. We divide the shuffled data into 10 parts. Then, 1 part was separated to reduce the risk of overfitting the training data, and 1 part was separated for performance evaluation. In total, 8 out of 10 parts were used for machine learning algorithm training [35]. The process is iterated for 10 times, traversing each part for validation. The method allows us to internally validate the performance of the prediction engine. K (=10) sets of results were compared across the algorithms.

### Outcomes

Hourly data were generated during the preprocessing step. For the outcome variable, the activity data for 3 hours were merged. If the participant walked during the 3 hours, the outcome was assigned as “walked.”

### Predictor Variables

In addition to 5 weeks’ hourly walking data, the variables noting the current date and time were used as predictors (Textbox 1). Each variable was encoded by the “One-hot-encoding” method [36]. It was a commonly used method to represent categorical (including ordinal or finite scale) variables in machine learning. The method converts the categorical variables (ie, N possible options) into an N-dimensional vector. Integers such as a current hour or current month were also converted into vectors. Each element of the vector can be ones or zeros. Each position in the vector denotes a particular value of options, and if a certain position was 1, the original value was mapped correspondingly. In a single vector, only one “1” was allowed. Since the encoding method enables the machine learning algorithm to train fast, it was commonly used. The discussion on the impact of the method on prediction performance was inconclusive [36].

**Variables used in classification algorithms.**

**Predictor variables**

- Current hour (24 dichotomous variables, one-hot-encoded)
- Today’s day of the week (7 dichotomous variables, one-hot-encoded)
- Current month (12 dichotomous variables, one-hot-encoded)
- Current day of the month (31 dichotomous variables, one-hot-encoded)
- Five Weeks’ hourly walking (Yes/No/Missing, 3 dichotomous variables, one-hot-encoded)

**Outcome variable**

- Whether the individual will walk during the next 3 hours (Yes/No, 1 dichotomous variable)

### Random Search for Multilayered Perceptron Model Structure

**Figure 3:**
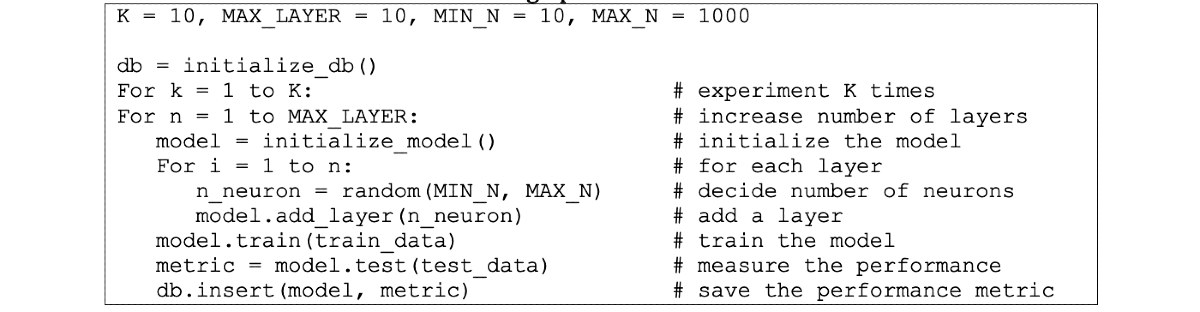
Pseudocode for searching optimal model structure.

Unlike other algorithms in this study, the multilayered perceptron (MLP) algorithm uses layer architectures as one of the critical performance factors. Optimization techniques such as evolutionary programming [37] or random search or grid search [38] may be used. A random search was used to minimize the implementation burden while not losing too much performance (Figure 3).

### Validation of the Models

The internal validation was performed by the K-fold validation methods. We used K=10. Individual test results were used to calculate the performance metrics such as accuracy, specificity, sensitivity, or MCCs. Data separation for the K-fold validation was conducted beforehand, which allows us to compare the metrics across the algorithms.

### Mathew Correlation Coefficient

MCC [39] was defined as follows:



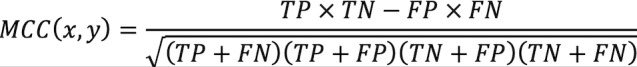



Where TP is true positive, TN is true negative, FP is false positive, and FN is false negative.

MCC was sometimes used as an optimization metric. In this study, we measured MCCs as a performance metric, not the optimization metric. Since we have balanced the output (see the Target Imbalance section), accuracy was used as the optimization metric.

### Computation Time

To conduct fair comparisons for the computation time, each model was trained in an isolated, standardized computing environment so that the system clock could measure the time elapsed. The system was reset every time a single execution was completed to minimize the fallout of the previous execution to the upcoming execution. Elapsed times were averaged and analyzed per algorithm.

## Results

### Study Population and Baseline Characteristics

A total of 41 (93%) out of 44 participants were included in the analysis [23]. The population's average age was 35.9 years. Of the 44 study participants, 31 (71%) were female, 26 (59%) were White, and 13 (30%) were Asian, with 36 (82%) having college degree or more. Moreover, 27% (n=12) of the participants had used a fitness app or activity tracker (Table 1).

**Table 1:** Baseline characteristics of participants at study entry.

Variable		Value
**Gender, n (%)**		
	Female	31 (71)
	Male	13 (30)
**Race, n (%)**		
	White	26 (59)
	Asian	13 (30)
	Black or African American	2 (5)
	Other	3 (7)
**Education, n (%)**		
	Some college	8 (18)
	College degree	13 (30)
	Some graduate school or graduate degree	23 (52)
Married or in a domestic partnership, n (%)		15 (34)
Have children, n (%)		16 (36)
Used fitness app before HeartSteps, n (%)		12 (27)
Used activity tracker before HeartSteps, n (%)		10 (22)
**Phone used for study app, n (%)**		
	Used personal phone	21 (48)
	Used study-provided phone	23 (52)
Age (years), mean (SD)		35.9 (14.7)

### Data Summary for Predictor and Outcome Variables

**Figure 4:**
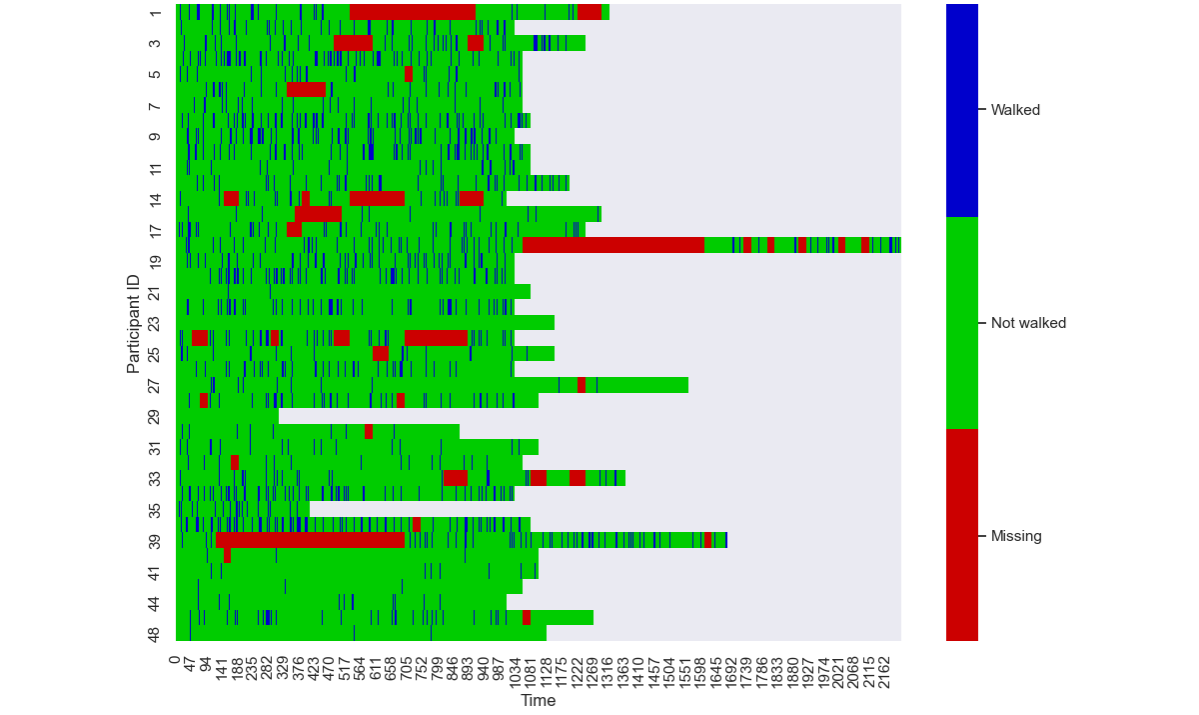
Overall distribution of walking data (1 narrow cell=1 hour).

On average, participants had available walking data for 43.3 (SD 9.1) days and 145.7 (SD 44.6) minutes per day. The average number of walking minutes per participant per day was reduced to 53.3 (SD 26.1) minutes after filtering with the threshold of 60 steps per minute (Methods section). Participants had 2.6 (SD 1.7) walks (ie, 5 or more consecutive walking minutes) every day (Methods section). Average length of each walk was 10.3 (SD 8.0) minutes. In hourly view, the participants had 0.6 (SD 0.1) “walking hours” (ie, the hours in which the participant walked) per day (Figure 4). Missing data were also used as a predictor state (Methods section). There were 18.1 (SD 13.4) missed days on average per participant, equivalent to 36.9% (SD 26.3%) of total days per participant. In the matter of outcome variable, as training and validating data set, 8129 “walking hours” and 37,711 “non-walking hours” (eg, nighttime or sedentary hours) were prepared (Methods section). Across the data, 17.7% of the time included participant activity. Thus, inactive time is 4.64 times more common than active time. The target imbalance was handled by undersampling (Methods section).

### Development of Prediction Algorithms

**Figure 5:**
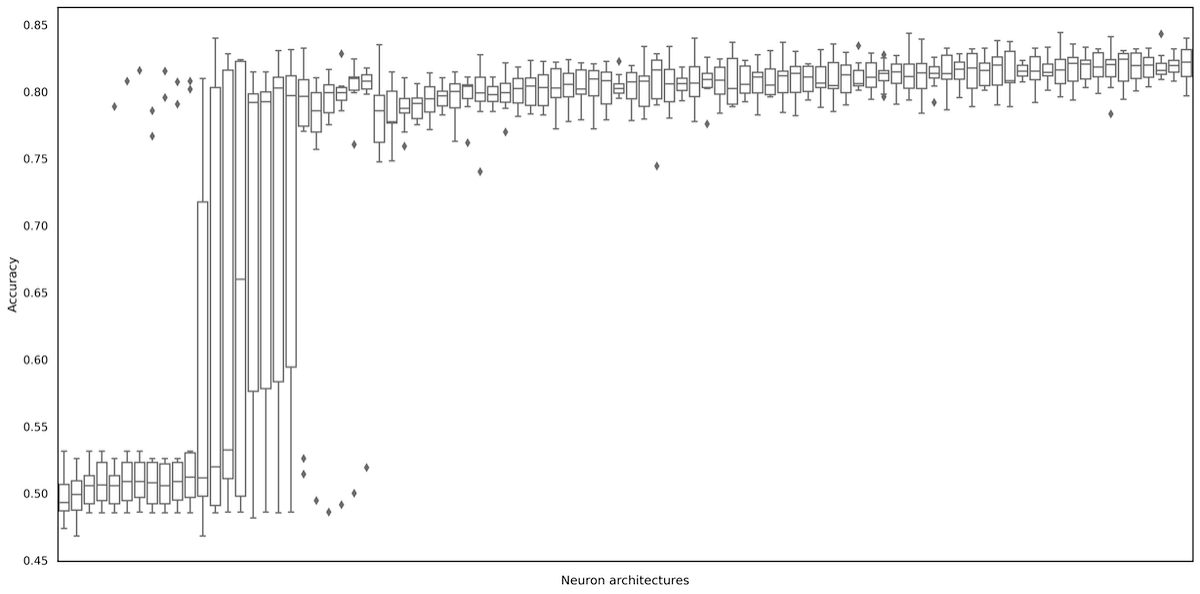
Performance of tried neuron architectures (90 trials).

The calculation time vastly varied (Table 2). The radial basis function support vector machine algorithm and multilayered perceptron algorithm took the longest period to run. Tree-based algorithms such as decision tree and random forests were shorter than others. Random search to discover the optimal layer structure was tried. The optimization process improved the accuracy of the MLP algorithms from 49.8% to 82.1%. The process also improved all other metrics (Figure 5).

**Table 2:** Performance metrics of tried algorithms.

Algorithms	Accuracy, mean (SD)	MCC^a^, mean (SD)	Sensitivity, mean (SD)	Specificity, mean (SD)
Logistic regression	0.772 (0.012)	0.545 (0.024)	0.795 (0.015)	0.749 (0.023)
RBF^b^ SVM^c^	0.693 (0.010)	0.389 (0.020)	0.746 (0.022)	0.641 (0.017)
XGBoost	0.763 (0.015)	0.530 (0.030)	0.816 (0.010)	0.711 (0.030)
Multilayered perceptron	0.820 (0.011)	0.643 (0.021)	0.861 (0.030)	0.778 (0.033)
Decision tree	0.636 (0.015)	0.281 (0.026)	0.509 (0.075)	0.762 (0.049)
Random forest	0.695 (0.010)	0.396 (0.023)	0.776 (0.019)	0.614 (0.018)

^a^MCC: Mathew correlation coefficient.

^b^RBF: radial basis function.

^c^SVM: support vector machine.

### Validation and Model Performance

**Figure 6:**
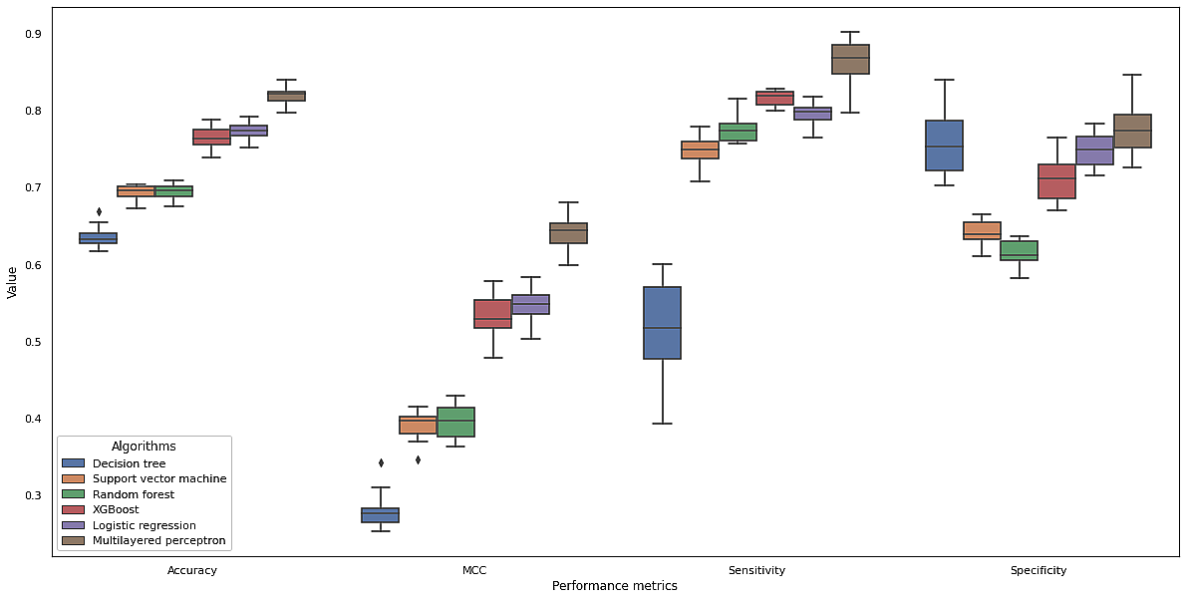
Performance metrics of the tried models. The top 3 architectures were chosen among multilayered perceptron engines. MCC: Mathew correlation coefficient.

The reference algorithm (logistic regression) showed 77.2% (SD 1.2%) accuracy. XGBoost showed 76.3% (SD 1.5%), radial basis function support vector machine showed 69.3% (SD 1.0%), decision tree showed 63.6% (SD 1.5%), and random forest showed 69.5% (SD 1.0%), respectively. MLP performance largely varied from 49.8% (SD 1.7%) to 82.1% (SD 1.3%). Only 3 MLP architectures with the highest accuracies were included (Tables 2 and 3; Figure 6). Sensitivities, specificities, and MCC showed similar patterns to the accuracies. The decision tree algorithm generally showed the lowest performance overall, except on the dimension of specificity. MLP showed the highest performance across metrics (82.0% accuracy, 86.1% sensitivity, and 77.8% specificity).

**Table 3:** Average confusion matrix of each model of K-fold validation for the validation data set.

	True positive, mean (SD)	True negative, mean (SD)	False positive, mean (SD)	False negative, mean (SD)
Logistic regression	646.3 (27.3)	609.0 (30.6)	203.5 (18.8)	166.2 (11.7)
RBF^a^ SVM^b^	606.3 (25.4)	520.3 (18.3)	292.2 (19.4)	206.2 (19.5)
XGBoost	663.0 (18.3)	577.6 (33.3)	234.9 (24.7)	149.5 (12.3)
MLP^c^	699.9 (35.2)	632.6 (34.7)	180.0 (27.5)	112.6 (24.2)
Decision tree	413.8 (65.4)	619.7 (52.5)	192.8 (39.1)	398.7 (56.5)
Random forest	630.3 (13.6)	499.0 (18.2)	313.5 (20.9)	182.2 (20.7)

^a^RBF: radial basis function.

^b^SVM: support vector machine.

^c^MLP: multilayered perceptron.

### Computation Time

In all the tested performance indicators, the optimized MLP showed the best performance and showed the second-longest training time of 225 seconds on average (Table 4). If we add up the total training time of all 90 optimization experiments, it took 56 hours. It was feasible to consistently evaluate training speed, accuracy, MCC, sensitivity, and specificity within the standardized performance evaluation framework. Through 90 random experiments, multiple MLP algorithms with optimized performance were obtained. The development, validation, and evaluation protocols can be used for similar prediction or classification problems.

Python 3.7.3, Sci-Kit Learn 1.0.2, Numpy 1.21.6, and Pandas 1.3.5, Tensorflow 2.8.0, xgboost 0.90, keras 2.8.0 were used.

**Figure 7:**
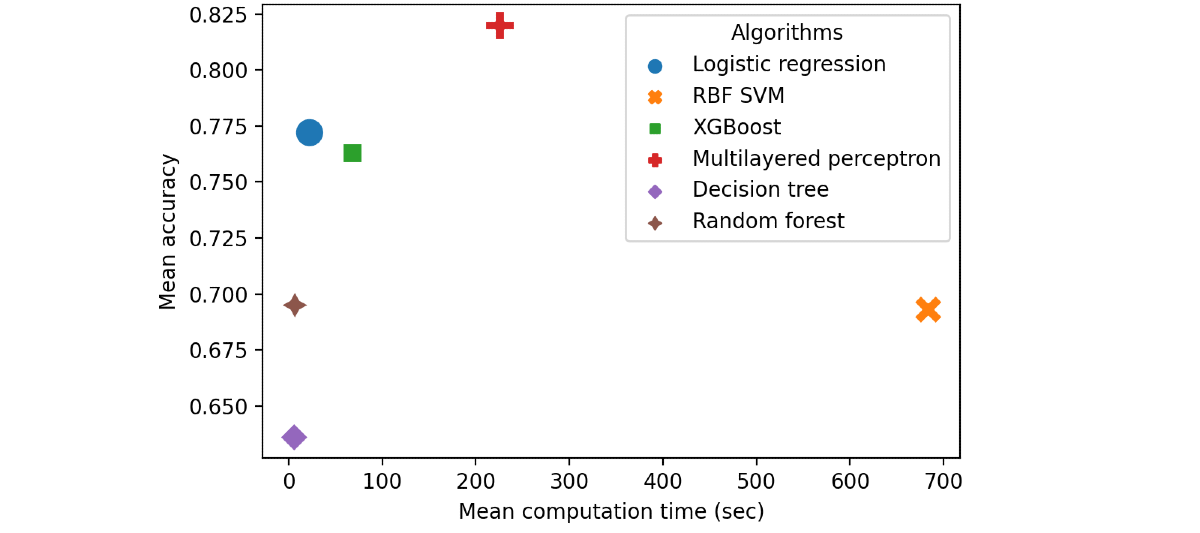
The comparisons between algorithms in the matter of mean computation time and mean prediction accuracy. RBF: radial basis function; SVM: support vector machine.

In the matter of computation cost-efficiency (ie, predictive performance vs computation time), each algorithm showed characteristic results. The logistic regression had reasonable prediction performance and relatively low average computation time cost, whereas MLP showed generally higher prediction performance but had the second highest average computation cost (Figure 7).

**Figure 8:**
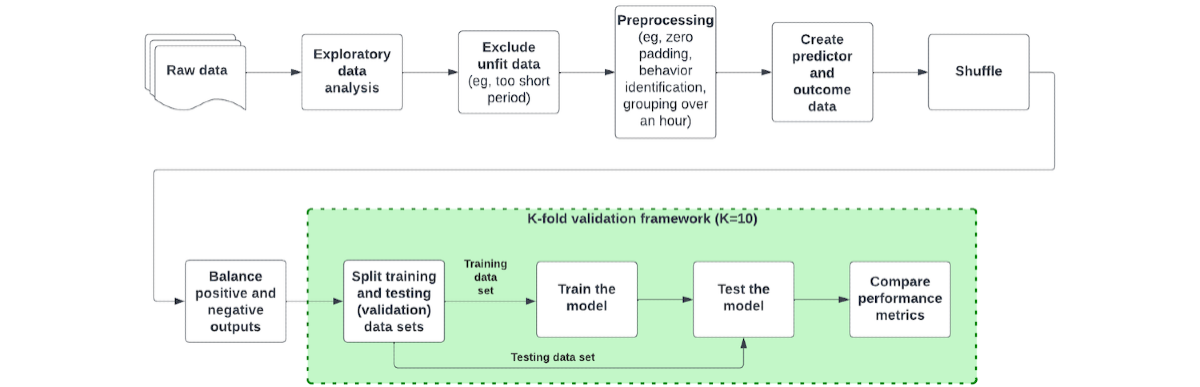
The data processing protocol.

It was feasible to consistently evaluate training speed, accuracy, MCC, sensitivity, and specificity within the standardized performance evaluation framework. Through 90 random experiments, multiple MLP algorithms with optimized performance were obtained. The development, validation, and evaluation protocols can be used for similar prediction or classification problems (Figure 8).

**Table 4:** Computation time to reach optimally trained status (seconds^a^).

Algorithms	Minimum	Maximum	Mean (SD)	CI
Logistic regression	20.73	24.89	22.37 (1.50)	19.43-25.31
RBF^b^ SVM^c^	413.09	683.62	496.57 (94.58)	311.19-681.96
XGBoost	63.92	73.75	67.79 (4.33)	59.30-76.27
Multilayered perceptron	172.14	300.36	225.35 (38.83)	149.24-301.46
Decision tree	3.30	13.20	5.89 (2.68)	0.65-11.14
Random forest	4.32	13.42	6.63 (2.53)	1.68-11.57

^a^Computation was done in Google Colaboratory Pro+ (High-RAM mode with GPU hardware accelerator); 8 cores of Intel Xeon CPU 2.00 GHz, 53.4GB Memory, Tesla P100-PCIE-16GB.

^b^RBF: radial basis function.

^c^SVM: support vector machine.

## Discussion

### Key Implications

The high-level focus of our work is to develop approaches for using data from individuals themselves to create more individualized and adaptive support via digital technologies. In this paper, our goal was to test if predictive models could be generated that would be useful in terms of sensitive and specific probability estimates of the likelihood that someone will walk within an upcoming 3-hour window and that it could be done in a computationally efficient fashion. The latter part is important as computational efficiency is needed to enable the predictive models to be incorporated into future just-in-time adaptive interventions (JITAIs) that could use these predictive models to guide future decision-making. To support robust, automated decision-making within a JITAI to increase walking, our goal was to test if it would be feasible to produce predictive models that are informative for individuals in terms of identifying moments when a person has some chance of walking as opposed to either times when a person will clearly walk and thus does not need support, or times when there was near-zero probability that, in a given 3-hour window, a person will walk. If a predictive model could be produced that would provide this information, it would enable a JITAI that could incorporate these individualized predictions as a signal that could be used for making decisions on whether a given moment would be a *just-in-time* moment to provide a suggestion to go for a walk, with the predictive model used to predict the likelihood that, within the next 3 hours, the person would have the *opportunity* to walk while also having a need for a suggestion (ie, a person would not need a suggestion to walk if they are very likely to walk anyway). Our results, overall, suggest it is possible to generate said models in a scalable fashion, which could then be incorporated into a future JITAI that incorporates these individualized predictive models. Central to this work, the models produced here are definitionally idiographic in nature and thus appropriate for each individual. Thus, the results from the model should not be generalized to other samples. Instead, the key transportable knowledge from this work is the overall approach used for selecting models to guide individualized decision-making in future JITAIs (Figure 8).

### Principal Findings

We developed 6 models (one of which was a group of models, and we chose the best 3 model architectures) for predicting future walking behavior within the subsequent 3-hour period using the previous 5 weeks’ hourly walking data. MLP algorithm showed the best performance across all 4 metrics within this sample. A random search for MLP architecture produced an optimal model with the best performance. Using predictive engines to decide how to configure JITAIs could enable the mobile physical activity app to deliver more timely, appropriate intervention components such as in-app notifications. To the best of our knowledge, interventions that use predictive models to adjust to participant’s behavior are still uncommon. Thus, our study makes a significant contribution by introducing the use of predictive algorithms for optimizing JITAIs.

### Methodological Considerations and Comparison With Prior Work

In this study, we designed a protocol to develop and validate a predictive model for walking behavior. While developing the model, we had a few common issues that should be handled as follows.

#### Small Data Sets and the Potential Risk for Low External Validity

Despite the effort to validate the model with the K-fold cross-validation, since we are using a small number of short time-series data, high levels of external validity are not assumed. However, since the model we developed in this study did not assume any prior knowledge or variability (ie, nonparametric), additional training data are theorized to harness better performance. The model also did not use the pretrained coefficients; we used randomized coefficients. This leaves room for better performance and higher computation efficiency when we use the pretrained model from this study to extend the training. Publicly available lifestyle data, including the All-of-Us project [40] and the ones available on the public data platforms [41], will be a good way to extend the data set.

#### Target Imbalance

Target imbalance is defined as a significantly unequal distribution between the classes [33]. In numerous clinical [42,43] and behavioral [33] data modeling studies, target imbalance is a common issue. Although a few oversampling methodologies to tackle unbalanced output data have been developed [44], this study used an undersampling approach due to potential concerns of exaggerated accuracy [34]. The separate analysis with oversampling of the same data and methodologies showed 5%-10% increases in the accuracy. It is suspected that the underlying individual behavior patterns in the training samples are partly included in the test and validation samples.

#### Performance Metrics

Accuracy is the most commonly used performance metric to evaluate classification algorithms. However, the *accuracy* metric is also known to have the inability to distinguish between type 1 and type 2 errors [45]. The metrics of sensitivity and specificity are also commonly used to overcome the limitation of accuracy. The information represented by both metrics is partial (ie, both are addressing either type of error). MCC [46] is used more commonly in recent publications due to its statistical robustness against target imbalance, which is a common issue of clinical and behavioral data. Considering the imbalance of the classification problem of interest, we included MCC as a performance metric.

### Limitations of This Study

The original study was designed for the purpose of pilot-testing and demonstrating the potential of microrandomized trials. Thus, these analyses are all secondary in nature. Further, the initial study was a small study, with only a minimum amount of data (n=41) used. Additionally, since the participants were recruited in a homogeneous environment and demographic groups, the external validity of the algorithms may be limited. With that said, the overall approach for formulating predictive models and their selection could feasibly be used in the future and, thus, it is more of our protocol and approach that is likely to be generalizable and generally useful for JITAIs compared to any specific insights from the models we ran. We contend that, for any targeted JITAI, a precondition for this type of approach is the appropriate data available, and that, for any JITAI, it is more valuable to build algorithms that match localized needs and contexts than seek to take insights from some previous samples that are different from a target population and assume they will readily translate. This, of course, can be done with careful tests of transportability using strategies such as directed acyclic graphs to guide the production of estimands [47] that would create formalized hypotheses of transportability. However, this is a much higher bar for transportability that, while valuable, can often be prohibitive for fostering progress in JITAIs. Within our proposed approach, the strategy involves gleaning *good enough* data to enable a localized prediction algorithm appropriate for the targeted population to be produced, with subsequent deployment factoring in strategies and approaches for updating and improving the algorithms as new insights emerge.

### Implication and Future Work

The results of our study show that prediction algorithms can be used to predict future walking behavior in a fashion that can be incorporated into a future walking JITAI. In this study, we modeled without contextual information other than the date, time, or day of the week. However, if the machine learning algorithm is trained using the other contextual information such as intervention data (eg, whether the in-app notification message is sent or not, which type of message is sent, and which sentiment is used to draw attention), the prediction engine would be capable of simulating how the intervention components might change the behavior in the multiple hypothetical scenarios. This capability would enable us to use the prediction algorithms uniquely, that is, comparing two or more possible scenarios to decide the optimal intervention mode of a JITAI. We could decide whether to send a message, which message should be sent, or what sentiment we could use to draw attention to our intervention. A pragmatic study that assesses the efficacy of such an approach is necessary.

The search methods for the optimal architectures of MLP could be improved. Evolutionary programming [48] and weight-agnostic neural network [37] are promising approaches. Such improvement could find the MLP architectures’ better performance in shorter computation time.

### Conclusion

The protocol for developing and validating a prediction engine for health behavior was developed. As a case study, walking behavior classification models were developed and validated. MLP showed the highest overall performance of all tried algorithms, yet it needed relatively higher computation time. A random search for optimal layer structure was a promising approach for prediction engine development.

## Appendix

Multimedia Appendix 1 TRIPOD (Transparent Reporting of a multivariable prediction model for Individual Prognosis or Diagnosis) checklist: prediction model development and validation.

## Abbreviations

JITAI: just-in-time adaptive intervention
MCC: Mathew correlation coefficient
MLP: multilayered perceptron
PA: physical activity
TRIPOD: Transparent Reporting of a multivariable prediction model for Individual Prognosis or Diagnosis
